# Takotsubo Cardiomyopathy in Two Patients without Any Cardiac Symptom on Maintenance Hemodialysis

**DOI:** 10.1155/2013/640976

**Published:** 2013-09-12

**Authors:** Jun Muratsu, Atsuyuki Morishima, Hiroyasu Ueda, Hisatoyo Hiraoka, Katsuhiko Sakaguchi

**Affiliations:** ^1^Department of Nephrology and Hypertension, Sumitomo Hospital, 5-3-20 Nakanoshima, Kita-ku, Osaka 530-0005, Japan; ^2^Department of Cardiovascular Medicine, Sumitomo Hospital, 5-3-20 Nakanoshima, Kita-ku, Osaka 530-0005, Japan

## Abstract

Takotsubo cardiomyopathy is a disorder characterized by left ventricular apical ballooning and electrocardiographic changes in the absence of coronary artery disease. While reversible in many cases, the mechanism of this disorder remains unclear. The most frequent clinical symptoms of takotsubo cardiomyopathy on admission are chest pain and dyspnea, resembling acute myocardial infarction. Here, we describe two cases of takotsubo cardiomyopathy without chest pain or dyspnea in patients on maintenance hemodialysis. The asymptomatic nature of these two cases may be due to the patients being on hemodialysis. Periodic electrocardiograms (ECG) may be helpful in screening this population for asymptomatic takotsubo cardiomyopathy and in evaluating its incidence.

## 1. Introduction

Takotsubo cardiomyopathy, derived from the Japanese term for “octopus pot,” is an unusual form of acute cardiomyopathy showing left ventricular apical ballooning with a distinct neck, a shape that mimics traps used to catch octopus, and is often triggered by intense physical or emotional distress [1]. Although maintenance hemodialysis patients usually have either or both extra physical or emotional stress [2], it is noteworthy that cases of takotsubo cardiomyopathy have been rarely reported previously in this population. We describe two cases of takotsubo cardiomyopathy in hemodialysis patients.

## 2. Case Reports

### 2.1. Case 1

**Figure 1:**
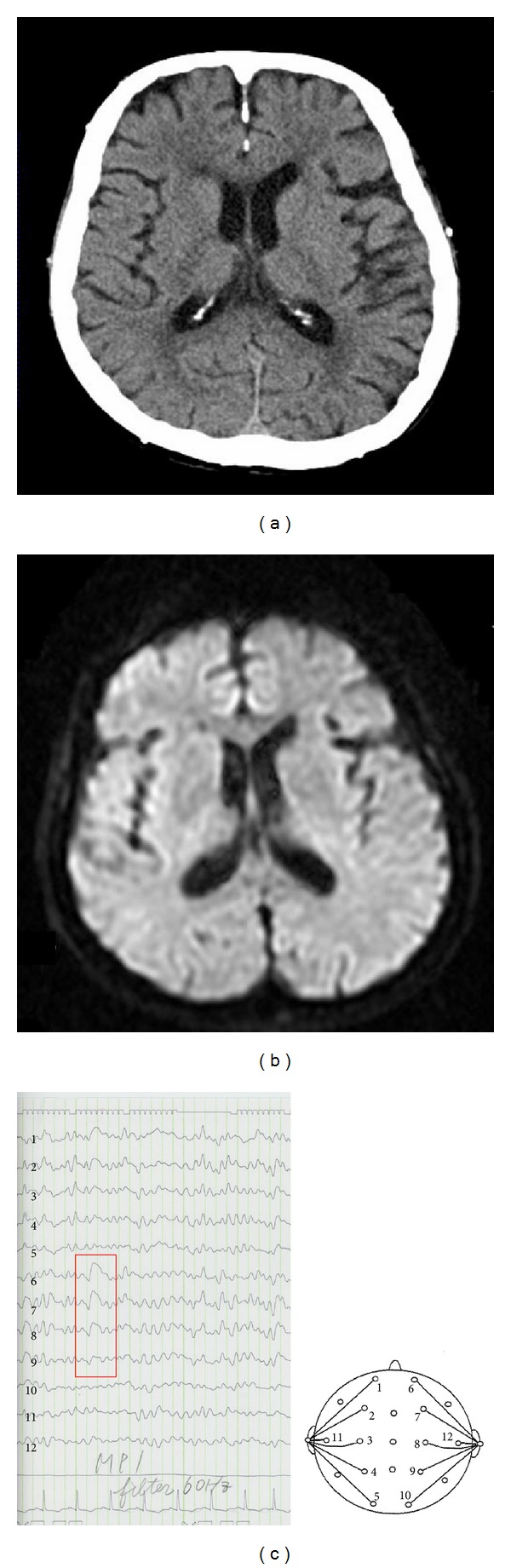
Case 1. Brain computed tomography (CT) and magnetic resonance imaging (MRI) on admission. Brain CT showed no acute cerebral bleeding (a). Brain MRI showed no acute cerebral infarction (b). Electroencephalography after admission showed repetition of intermittent high-amplitude irregular slow waves in the right frontal lobe (c). *Lead placement: 1, left frontal pole (Fp1); 2, left frontal (F3); 3, left central (C3); 4, left parietal (P3); 5, left occipital (O1); 6, right frontal pole (Fp2); 7, right frontal (F4); 8, right central (C4); 9, right parietal (P4); 10, right occipital (O2); 11, left mid temporal (T3); 12, right mid temporal (T4).

A 63-year-old female on maintenance hemodialysis was admitted to our hospital for an initial generalized tonic seizure suffered at home. Just after admission, a second generalized tonic seizure was observed. During the seizure, conjugate eye deviation toward the upper left was noted. On admission, her pulse rate was 92 beats/min, blood pressure 134/92 mm Hg, and body temperature 36.5°C. Neither abnormal heart sounds nor rales were noted on auscultation. Brain computed tomography (CT) and magnetic resonance imaging (MRI) showed no mass lesion, hemorrhage, or infarction (Figures 1(a) and 1(b)). Electroencephalography showed repetition of intermittent high-amplitude irregular slow waves in the right frontal lobe (Figure 1(c)). Based on these findings, the patient was diagnosed with generalized partial seizures. After administration of phenytoin sodium, the seizures resolved, and neurological findings normalized. According to her medical record, she had no history of diabetes mellitus or coronary artery disease, nor any family history of coronary artery disease. She had been stable on maintenance hemodialysis for 32 years, and the appropriate dry weight was maintained. Kt/V (urea) was 1.48. Her administration had included fentanyl patch, etizolam, rabeprazole sodium, celecoxib, lactomin, camostat mesilate, tocopherol nicotinate, acetaminophen, and mecobalamin. On admission, electrocardiogram (ECG) was normal.

**Figure 2:**
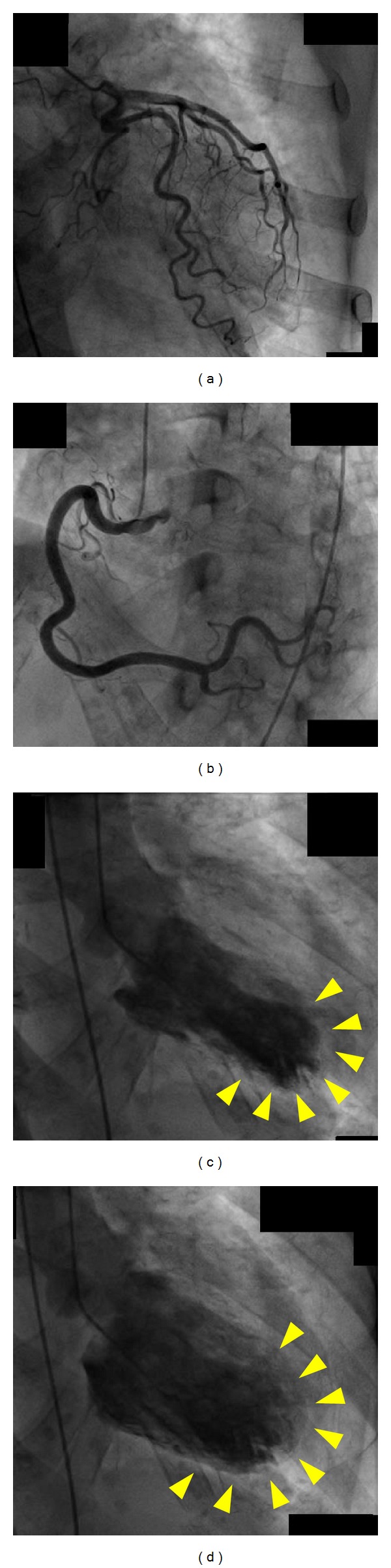
Case 1. Coronary angiography and left ventriculography were performed on the second hospital day. Coronary angiography showed normal right (a) and left (b) coronary arteries and branches. Left ventriculography showed akinesis of the left ventricle apex (arrowheads). (c) End-systolic phase; (d) end-diastolic phase.

On the second hospital day, ECG showed inverted T waves and QT prolongation in all leads without chest pain, dyspnea, or any other cardiac symptoms. Neurological findings were normal. Blood test findings were as follows: creatinine, 6.46 mg/dL; blood urea nitrogen, 55 mg/dL; sodium, 141 mEq/L; potassium, 5.3 mEq/L; calcium, 8.9 g/dL; phosphorus, 7.0 mg/dL; hemoglobin, 12.3 g/dL; aspartate aminotransferase, 19 IU/L; alanine aminotransferase, 8 IU/L; creatine kinase, 312 IU/L; and cardiac troponin *t*-test, negative (Troponin T kit; TROP T sensitive, Roche Diagnostics, Mannheim, Germany: cutoff value is 0.1 ng/mL). Thoracoabdominal computed tomography (CT) did not show any abnormal findings. Echocardiography demonstrated left apical akinesis. On the third hospital day, the patient underwent cardiac catheterization. While coronary angiography showed normal coronary arteries, left ventriculography showed extensive severe hypokinesis in the anteroseptal and apical segments with hyperkinesis in the basal segments (Figure 2). Based on these findings, the patient was diagnosed with takotsubo cardiomyopathy. 

**Figure 3:**
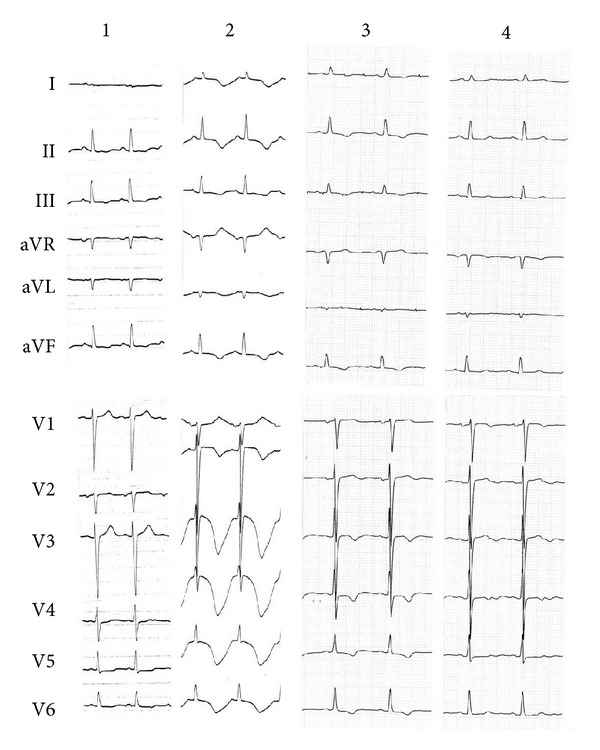
Case 1. Electrocardiographic changes, at admission (1), day 2 (2), day 20 (3), and day 62 (4).

We followed with serial ECG monitoring for several weeks. Her clinical course of takotsubo cardiomyopathy was uneventful and seizure had not been seen. After two months, left ventricular apical wall motion abnormalities on echocardiography had reversed, and the inverted T waves and QT prolongation on ECG had also resolved (Figure 3). 

### 2.2. Case 2

**Figure 4:**
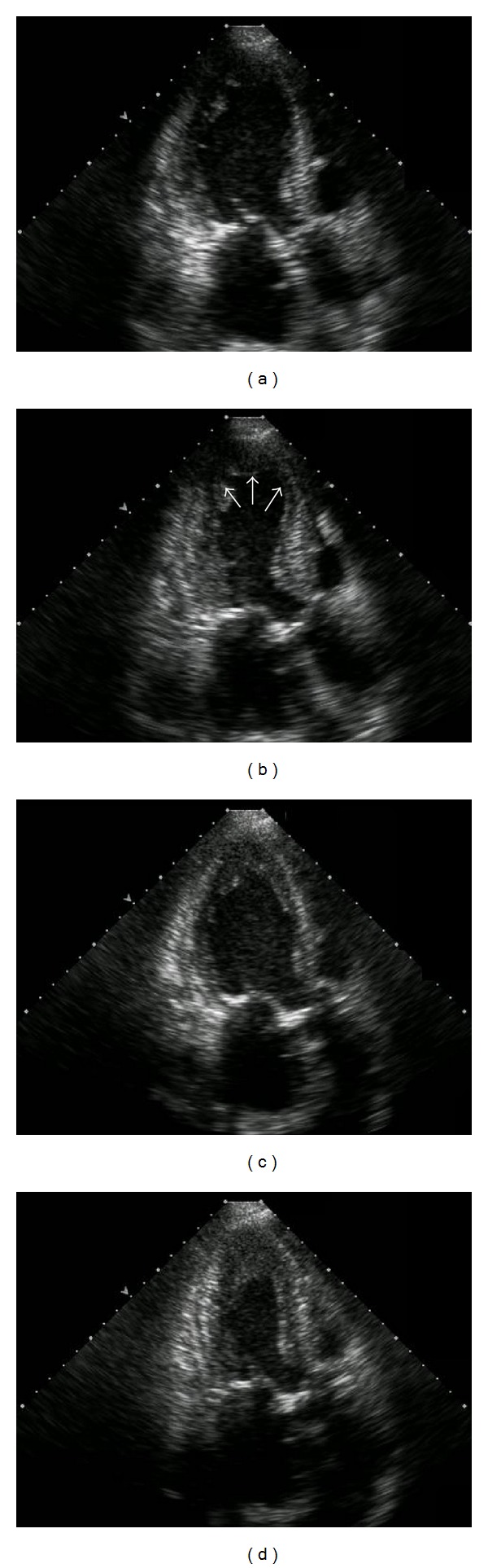
Case 2. ((a), (b)) Echocardiogram on admission demonstrated apical akinesis and ballooning of the left ventricle apex (arrows). (a) End-diastolic phase; (b) end-systolic phase. ((c), (d)) Echocardiogram 3 weeks later showed recovered left ventricular apical wall motion (c) End-diastolic phase; (d) end-systolic phase.

A 59-year-old female on maintenance hemodialysis visited the hemodialysis clinic for fatigue. The patient had had glomerulonephritis and had been on hemodialysis for 12 years. She had no history of diabetes mellitus or coronary artery disease, nor any family history of coronary artery disease. She had been charged with caring for her daughter with an acute illness two days prior to admission. Routine ECG showed giant inverted T waves in leads V1–V5, and blood tests revealed a creatinine level of 8.04 mg/dL, blood urea nitrogen of 32 mg/dL, sodium of 140 mEq/L, potassium of 4.4 mEq/L hemoglobin of 10.2 g/dL, aspartate aminotransferase of 29 IU/L, alanine aminotransferase of 18 IU/L, creatine kinase level of 129 IU/L, and cardiac troponin I level of 0.107 ng/mL (normal range: 0.00–0.10 ng/mL). The patient was referred to our hospital because of the ECG abnormalities; she had no complaints of chest pain or dyspnea. On admission to our hospital, her pulse rate was 82 beats/min, blood pressure 134/80 mm Hg, and body temperature 36.9°C. She was alert; the bulbar conjunctivae were not icteric, and the palpebral conjunctivae were not pale. Normal respiratory and heart sounds were noted on auscultation. Neurological findings were also normal. Abdominal ultrasonography did not show any abnormal findings. According to her medical records, she had been quite stable on maintenance hemodialysis, and an appropriate dry weight had been maintained. Kt/V (urea) was 1.38. Her administration had included lanthanum carbonate hydrate, telmisartan, teprenone, pravastatin sodium, and cinacalcet hydrochloride. Echocardiography demonstrated akinesis in the apical segment of the left ventricle (Figures 4(a) and 4(b)). While coronary angiography showed normal coronary arteries, left ventriculography showed extensive severe anteroseptal and apical hypokinesis with hyperkinesis of the basal segments. Based on these findings, the patient was diagnosed with takotsubo cardiomyopathy. A follow-up echocardiogram three weeks later showed dramatic improvement in the apical wall motion without any specific treatment (Figures 4(c) and 4(d)).

## 3. Discussion

We described two cases of takotsubo cardiomyopathy in patients without any cardiac disease-associated symptom on maintenance hemodialysis. Takotsubo cardiomyopathy has been characterized by left ventricular apical ballooning, electrocardiographic changes without coronary artery disease, and improvement within weeks in most cases [1]. In 2004, researchers at the Mayo Clinic proposed the following diagnostic criteria, which were subsequently modified in 2008: transient hypokinesis, akinesis, or dyskinesis in the left ventricular mid segments with or without apical involvement; regional wall motion abnormalities that extend beyond a single epicardial vascular distribution; presence of a stressful trigger in most (but not all) cases; absence of obstructive coronary disease or angiographic evidence of acute plaque rupture; new ECG abnormalities (ST-segment elevation and/or T-wave inversion); modest elevation in cardiac troponin; and absence of pheochromocytoma or myocarditis [3]. 

While the exact pathophysiological basis of the distinctive contractile pattern in takotsubo cardiomyopathy remains to be elucidated, several suspected mechanisms have been reported so far [4], one of which is catecholamine cardiotoxicity [5]. High plasma catecholamine levels in patients with pheochromocytoma are well known to induce reversible cardiomyopathy [6], and plasma levels of both epinephrine and norepinephrine are markedly increased at the onset of takotsubo cardiomyopathy. Wittstein et al. suggested that markedly elevated catecholamine levels might be the main pathogenetic factor [7]. It is reported that sympathetic nervous system activity is inappropriately increased in hemodialysis patients [8, 9]. Long-term maintenance hemodialysis patients may be predisposed to catecholamine cardiotoxicity developing takotsubo cardiomyopathy. Several recent reports have suggested that takotsubo cardiomyopathy is directly or indirectly linked with an inappropriate release of antidiuretic hormone (ADH) [10]. Indeed, seizures, solid tumors such as lymphoma and metastatic neoplasms, asthma, SSRIs, carbamazepine, and NSAIDs are all known causes of an inappropriate release of ADH, and all have been previously reported as triggers of takotsubo cardiomyopathy [11–16]. In chronic hemodialysis patients, plasma ADH levels are significantly higher than those in normal subjects [17, 18]. We did not measure the plasma catecholamine levels and ADH levels in the present cases at the onset of takotsubo cardiomyopathy and have no evidence regarding the causal relationship between takotsubo cardiomyopathy and high ADH level. However, it seemed sensible to assume that high ADH level in addition to catecholamine level might develop serious cardiotoxicity such as takotsubo cardiomyopathy on maintenance hemodialysis. Further study should be required. 

Patients on maintenance hemodialysis have a well-known increased risk of cardiovascular morbidity and mortality, usually from coronary artery disease [19]. On the other hand, in epidemiologic studies, some cases of acute renal failure were reported; however, only three cases of takotsubo cardiomyopathy in maintenance hemodialysis patients have been described so far [1, 7, 20–29]. We list the known cases of takotsubo cardiomyopathy in patients on maintenance hemodialysis in Table 1 [27–29]. Factors as a trigger for takotsubo cardiomyopathy are various. In our cases, they included new-onset seizures and reaction to a family member's acute illness. A number of complications associated with maintenance hemodialysis, such as infection, cardiovascular dysfunction, cerebrovascular accident, and as, is our cases, epileptic seizure and excessive worry about illness, could also be the trigger onset of takotsubo cardiomyopathy [1, 30]. Most cases reported so far have been in middle-aged females on long-term dialysis, with glomerulonephritis as the cause of renal failure. The relationship between glomerulonephritis and takotsubo cardiomyopathy has not been clarified. Further experience is needed. Hemodialysis is associated with cardiovascular dysfunction, anemia, malnutrition, muscle wasting, muscle weakness, neuropathy, glucose intolerance, and reduced bone density [31]. As a consequence, hemodialysis impairs quality of life (QOL) [32], and hemodialysis patients are under physical or emotional stress [2]. It has been found that about 50% of hemodialysis patients suffer from depression. The major factors for depression in hemodialysis patients include female gender and longer time on dialysis [33]. Takotsubo cardiomyopathy occurs predominantly in postmenopausal women after exposure to emotional or physical stress. From the point of view that postmenopausal women after exposure to emotional or physical stress are likely to develop takotsubo cardiomyopathy, we should pay considerable attention to middle-aged long-term hemodialysis women. 

As mentioned above, takotsubo cardiomyopathy on maintenance hemodialysis is rarely reported. While the most frequent clinical symptoms of takotsubo cardiomyopathy on admission are chest pain and dyspnea, resembling acute myocardial infarction [4, 34], the two patients described here had no complaints of severe chest pain, chest discomfort, or dyspnea and were instead diagnosed based on ST-segment abnormalities by ECG recordings. As such, if electrocardiographic monitoring had not been performed while they were in hospital, the cardiomyopathy may have been missed. Our cases indicate that takotsubo cardiomyopathy in maintenance hemodialysis patients may develop with fewer symptoms. It is a very important clinical problem and should be clarified in the future whether many takotsubo cardiomyopathy cases who have renal failure on maintenance hemodialysis may be overlooked or not. We suggest that ECG monitoring should be performed at the time of acute events associated with hemodialysis in patients even if they have no cardiac symptoms. The prognosis of patients with takotsubo cardiomyopathy is generally favorable; however, complications occur in about 20% of cases, and a case of left ventricular free wall rupture and death associated with takotsubo cardiomyopathy has been reported [11, 35, 36]. We should close-observe patients for several weeks. 

Our observations in these two cases suggest that takotsubo cardiomyopathy in hemodialysis patients can be asymptomatic and may be underdiagnosed. Further investigation is needed to assess the incidence and possible prevention of takotsubo cardiomyopathy in hemodialysis patients. 

## Figures and Tables

**Table 1:** Reports of takotsubo cardiomyopathy in hemodialysis patients.

Age	Gender	Duration of hemodialysis	Underlying kidney disease	Symptoms at onset	Factor as a trigger	References
84	F	2 years	Glomerulonephritis	Chest discomfort	Stopped smoking	[27]
61	F	20 years	Glomerulonephritis	Chest pain and dyspnea	Surgery for cervical spondylosis	[28]
65	F	9 years	Diabetic nephropathy	Severe left shoulder pain	Headache and fever up	[29]
63	F	32 years	Glomerulonephritis	None	Epileptic seizure	Case 1
59	F	12 years	Glomerulonephritis	Fatigue	Family acute illness	Case 2

## References

[1] Tsuchihashi K, Ueshima K, Uchida T (2001). Transient left ventricular apical ballooning without coronary artery stenosis: a novel heart syndrome mimicking acute myocardial infarction. *Journal of the American College of Cardiology*.

[2] Heiwe S, Clyne N, Dahlgren MA (2003). Living with chronic renal failure: patients’ experiences of their physical and functional capacity. *Physiotherapy Research International*.

[3] Prasad A, Lerman A, Rihal CS (2008). Apical ballooning syndrome (Tako-Tsubo or stress cardiomyopathy): a mimic of acute myocardial infarction. *American Heart Journal*.

[4] Akashi YJ, Goldstein DS, Barbara G, Ueyama T (2008). Takotsubo cardiomyopathy a new form of acute, reversible heart failure. *Circulation*.

[5] Kume T, Kawamoto T, Okura H (2008). Local release of catecholamines from the hearts of patients with tako-tsubo-like left ventricular dysfunction. *Circulation Journal*.

[6] Frustaci A, Loperfido F, Gentiloni N, Caldarulo M, Morgante E, Russo MA (1991). Catecholamine-induced cardiomyopathy in multiple endocrine neoplasia: a histologic, ultrastructural, and biochemical study. *Chest*.

[7] Wittstein IS, Thiemann DR, Lima JAC (2005). Neurohumoral features of myocardial stunning due to sudden emotional stress. *The New England Journal of Medicine*.

[8] Converse RL, Jacobsen TN, Toto RD (1992). Sympathetic overactivity in patients with chronic renal failure. *The New England Journal of Medicine*.

[9] Hausberg M, Kosch M, Harmelink P (2002). Sympathetic nerve activity in end-stage renal disease. *Circulation*.

[10] Falola M, Fonbah W, McGwin G (2012). Takotsubo cardiomyopathy versus ST-elevation myocardial infarction in a large case-control study: proposing a new mechanism. *International Journal of Cardiology*.

[11] Bybee KA, Kara T, Prasad A (2004). Systematic review: transient left ventricular apical ballooning: a syndrome that mimics ST-segment elevation myocardial infarction. *Annals of Internal Medicine*.

[12] Burgdorf C, Kurowski V, Bonnemeier H, Schunkert H, Radke PW (2008). Long-term prognosis of the transient left ventricular dysfunction syndrome (Tako-Tsubo cardiomyopathy): focus on malignancies. *European Journal of Heart Failure*.

[13] Santos M, Dias V, Meireles A (2011). Hyponatremia—an unusual trigger of Takotsubo cardiomyopathy. *Revista Portuguesa de Cardiologia*.

[14] Kawano H, Matsumoto Y, Arakawa S, Hayano M, Fijisawa H (2011). Takotsubo cardiomyopathy in a patient with severe hyponatremia associated with syndrome of inappropriate antidiuretic hormone. *Internal Medicine*.

[15] Abouezzeddine O, Prasad A (2010). Apical ballooning syndrome precipitated by hyponatremia. *International Journal of Cardiology*.

[16] Summers MR, Lennon RJ, Prasad A (2010). Pre-morbid psychiatric and cardiovascular diseases in apical ballooning syndrome (tako-tsubo/stress-induced cardiomyopathy). Potential pre-disposing factors?. *Journal of the American College of Cardiology*.

[17] Shimamoto K, Watarai I, Miyahara M (1977). A study of plasma vasopressin in patients undergoing chronic hemodialysis. *Journal of Clinical Endocrinology and Metabolism*.

[18] Nord E, Danovitch GM (1979). Vasopressin response in haemodialysis patients. *Proceedings of the European Dialysis and Transplant Association*.

[19] Sarnak MJ, Levey AS, Schoolwerth AC (2003). Kidney disease as a risk factor for development of cardiovascular disease: a statement from the american heart association councils on kidney in cardiovascular disease, high blood pressure research, clinical cardiology, and epidemiology and prevention. *Hypertension*.

[20] Kurisu S, Sato H, Kawagoe T (2002). Tako-tsubo—like left ventricular dysfunction with ST-segment elevation: a novel cardiac syndrome mimicking acute myocardial infarction. *American Heart Journal*.

[21] Akashi YJ, Musha H, Kida K (2005). Reversible ventricular dysfunction takotsubo cardiomyopathy. *European Journal of Heart Failure*.

[22] Bybee KA, Prasad A, Barsness GW (2004). Clinical characteristics and Thrombolysis in Myocardial Infarction frame counts in women with transient left ventricular apical ballooning syndrome. *American Journal of Cardiology*.

[23] Sharkey SW, Lesser JR, Zenovich AG (2005). Acute and reversible cardiomyopathy provoked by stress in women from the United States. *Circulation*.

[24] Kurowski V, Kaiser A, von Hof K (2007). Apical and midventricular transient left ventricular dysfunction syndrome (tako-tsubo cardiomyopathy): frequency, mechanisms, and prognosis. *Chest*.

[25] Inoue M, Shimizu M, Ino H (2005). Differentiation between patients with takotsubo cardiomyopathy and those with anterior acute myocardial infarction. *Circulation Journal*.

[26] Yoshida T, Hibino T, Kako N (2007). A pathophysiologic study of tako-tsubo cardiomyopathy with F-18 fluorodeoxyglucose positron emission tomography. *European Heart Journal*.

[27] Fukui M, Mori Y, Tsujimoto S (2006). “Takotsubo” cardiomyopathy in a maintenance hemodialysis patient. *Therapeutic Apheresis and Dialysis*.

[28] Takemoto F, Chihara N, Sawa N (2009). Takotsubo cardiomyopathy in a patient undergoing hemodialysis. *Kidney International*.

[29] Kusaba T, Sasaki H, Sakurada T (2004). Takotsubo cardiomyopathy thought to be induced by MRSA meningitis and cervical epidural abscess in a maintenance-hemodialysis patient: case report. *Japanese Journal of Nephrology*.

[30] Park J-H, Kang S-J, Song J-K (2005). Left ventricular apical ballooning due to severe physical stress in patients admitted to the medical ICU. *Chest*.

[31] Moattari M, Ebrahimi M, Sharifi N, Rouzbeh J (2012). The effect of empowerment on the self-efficacy, quality of life and clinical and laboratory indicators of patients treated with hemodialysis: a randomized controlled trial. *Health Qual Life Outcomes*.

[32] Merkus MP, Jager KJ, Dekker FW (1997). Quality of life in patients on chronic dialysis: self-assessment 3 months after the start of treatment. *American Journal of Kidney Diseases*.

[33] Raymond CB, Wazny LD, Honcharik PL (2008). Pharmacotherapeutic options for the treatment of depression in patients with chronic kidney disease. *Nephrology Nursing Journal*.

[34] Sato M, Fujita S, Saito A (2006). Increased incidence of transient left ventricular apical ballooning (so-called “Takotsubo” cardiomyopathy) after the Mid-Niigata Prefecture earthquake. *Circulation Journal*.

[35] Akashi YJ, Tejima T, Sakurada H (2004). Left ventricular rupture associated with takotsubo cardiomyopathy. *Mayo Clinic Proceedings*.

[36] Merchant EE, Johnson SW, Nguyen P, Kang C, Mallon WK (2008). Takotsubo cardiomyopathy: a case series and review of the literature. *Western Journal of Emergency Medicine*.

